# Functional status and spatial interaction of T cell subsets driven by specific tumor microenvironment correlate with recurrence of non-small cell lung cancer

**DOI:** 10.3389/fimmu.2022.1022638

**Published:** 2023-01-04

**Authors:** Liying Yang, Wei Zhang, Jujie Sun, Guanqun Yang, Siqi Cai, Fenghao Sun, Ligang Xing, Xiaorong Sun

**Affiliations:** ^1^ Department of Radiation Oncology, Shandong Cancer Hospital and Institute, Shandong First Medical University and Shandong Academy of Medical Sciences, Jinan, China; ^2^ Department of Pathology, Shandong Cancer Hospital and Institute First Medical University and Shandong Academy of Medical Science, Jinan, China; ^3^ Shandong University Cancer Center, Shandong University, Jinan, China; ^4^ Department of Nuclear Medicine, Shandong Cancer Hospital and Institute, Shandong First Medical University and Shandong Academy of Medical Sciences, Jinan, China

**Keywords:** CD4, CD8, functional status, spatial interaction, prognostic, lung cancer

## Abstract

**Background:**

The anti-tumoral or pro-tumoral roles of CD4^+^ and CD8^+^ T cells typify the complexity of T cell subsets function in cancer. In the non-small cell lung cancer (NSCLC), the density and topology of distinct T cell phenotypes at the tumor center (TC) versus the invasive margin (IM) are largely unknown. Here, we investigated T cell subsets density and distribution within TC and IM regions in NSCLC and its impact on the prognosis.

**Methods:**

We performed multiplex immunofluorescence using a tissue microarray of samples from 99 patients with locally advanced NSCLC to elucidate the distributions of tumor cell, T cell subpopulations (CD4/conventional CD4/regulatory CD4/CD8/cytotoxic CD8/pre-dysfunctional CD8/dysfunctional CD8), microvessel density (MVD), cancer-associated fibroblasts (CAFs) and hypoxia-inducible factor-1α (HIF-1α) in TC and IM tissues. Cell-to-cell nearest neighbor distances and interactions were analyzed using the phenoptrreports R package. Cox regression was used to evaluate the associations between T cell subsets density and proximity to tumor cells and recurrence-free survival (RFS). Correlations between different cell subsets were examined by Spearman’s or Kruskal-Wallis tests.

**Results:**

In the locally advanced NSCLC, the proportion of tumor cells and CAFs in IM is lower than in the TC, while MVD, CD4^+^, and CD8^+^ T lymphocytes were increased, and tumor cells were closer to T lymphocytes and their subsets. The density and proximity of CD4^+^ and CD8^+^ T cells in the TC and IM regions were not associated with RFS, but in the IM area, increased density of dysfunctional CD8 and closer regulatory CD4 to tumor cells were independent risk factors for recurrence (HR were 3.536 and 2.884, respectively), and were positively correlated with HIF-1α^+^CD8 (r = 0.41, *P* = 0.000) and CAFs (*P* = 0.017), respectively.s

**Conclusions:**

In locally advanced NSCLC, the functional status of T cells in the IM region is closely related to recurrence. The density of dysfunctional CD8 and the proximity of regulatory CD4 to tumor cells were independent risk factors for recurrence, and are positively correlated with the hypoxia response of CD8^+^ T cells and CAFs. Targeting hypoxia or CAFs is expected to further sensitize therapy.

## 1 Introduction

The tumor microenvironment (TME), consisting of tumor cells, immune cells, blood vessels, stroma cells, and other components, is closely associated with the development of NSCLC (1–3). T cells and their role in prognosis have been extensively studied, and their increased infiltration is considered to result in a better prognosis (4–9). Traditionally, T cells are classified as CD4^+^ and CD8^+^ T cells by a single protein marker, but their association with the prognosis of NSCLC is controversial (6, 9–14).

Single-cell sequencing had shown that CD4^+^ and CD8^+^ T cells had different functional statuses in NSCLC (15–17). The conventional (CD4^+^ Tcon) and regulatory CD4 (CD4^+^ Treg) had their functions; however, their impact on prognosis remains controversial (4, 6, 9, 12, 18–20). CD8 could gradually change from a pre-dysfunctional (CD8^+^ Tpre) to a dysfunctional state (CD8^+^ Tdys) in response to continuous stimulation by tumor antigens (15, 17). Results from TCGA database found that high CD8^+^ Tpre combined with low CD8^+^ Tdys significantly prolonged OS in lung adenocarcinoma patients (17), but the detailed histological analysis was not available because of the lack of single protein markers.

Recent advances in multiplex immunofluorescence (mIF) have enabled simultaneous detection of multiple protein targets in a single tissue section, providing unique insights for accurately identifying and quantifying the functional status of T cell subsets (21–23). At the same time, mIF-based spatial proteomic analysis can quantify cell-to-cell nearest neighbor distances (NND) and interactions. Quantifying the functional macrophage cell populations and their proximity to tumor cells could provide better prognostic information for NSCLC (24). However, the functional T cells and their proximity to tumor cells in NSCLC remain unclear.

Moreover, recent literatures suggested topologically distinct distribution of T cells within the TME (7, 25, 26). The CD8^+^ T cells density with survival was highly significant at IM in contrast with TC of lung cancer (27). However, distribution patterns of functional T cell subsets between TC and IM in NSCLC remain unexplored. Therefore, we used mIF to analyze locally advanced NSCLC relapse-associated TME and explore its relationship with hypoxia-inducible factor-1α (HIF-1α), microvascular density (MVD), and cancer-associated fibroblasts (CAFs) in order to guide individualized therapy.

## 2 Materials and methods

### 2.1 Patient cohort

A total of 139 patients with primary NSCLC underwent radical surgery between January 1, 2014, and August 31, 2018, at Shandong Cancer Hospital. Clinicopathological information was obtained from the medical records. None of the patients were treated with neoadjuvant therapy. Recurrence-free survival was defined as the interval between radical surgery and the first relapse or death or between surgery and the last observation for patients without relapse. The actual number of patients analyzed in the study was 99, and the detail of the inclusion and exclusion criteria was shown in **Supplementary Figure S1.**

### 2.2 Tissue microarrays (TMA)

Formalin-fixed paraffin-embedded (FFPE) histological sections were collected for TMA construction. TMA was performed using standard procedures (27, 28), representative areas were carefully selected and marked on a hematoxylin and eosin stain slide, and 1 mm cores were obtained from the FFPE blocks using a needle and inserted into a recipient paraffin block. All slides were examined by at least one experienced pathologist and four representative areas were selected: two TC regions and two IM regions. IM was defined as the region centered on the border separating the host tissue from the malignant nests, with an extent of 1 mm (25, 27, 29). Furthermore, we selected lymphoid tissue as a positive control (the core of lymphoid tissue was prepared for each tissue microarray), and the autofluorescence of the tissue was used as a negative control.

### 2.3 Multiplex immunofluorescence (mIF) staining

Two mIF panels were developed to characterize the locally advanced NSCLC immune microenvironment. CD4 panel including Pan-CK, CD4, FoxP3, CD8, CD31, α-SMA, HIF-1α and DAPI. CD8 panel including Pan-CK, CD8, CD103, PD-1, TIM-3 and DAPI.

The procedure for mIF has been previously described (21, 30–33). In summary, 3 μm serial sections obtained from TMA blocks were deparaffinized and rehydrated using a decreasing ethanol series. Antigen dretrieval was performed by using microwaves. A blocking buffer was then used to initiate protein stabilization and background reduction for 30-60 minutes at room temperature. Primary antibodies were applied and washed in 1×Tris-buffered saline containing 0.5% Tween 20. Isotype-specific HRP-conjugated antibodies and an Opal fluorophore (Akoya Biosciences). Finally, the nuclei were stained with DAPI (Akoya Biosciences) for 10 min. Antibodies and fluorophores used in the mIF procedures were detailed in **Supplementary Table 1**.

### 2.4 Multispectral imaging and spectral unmixing

**Figure 1:**
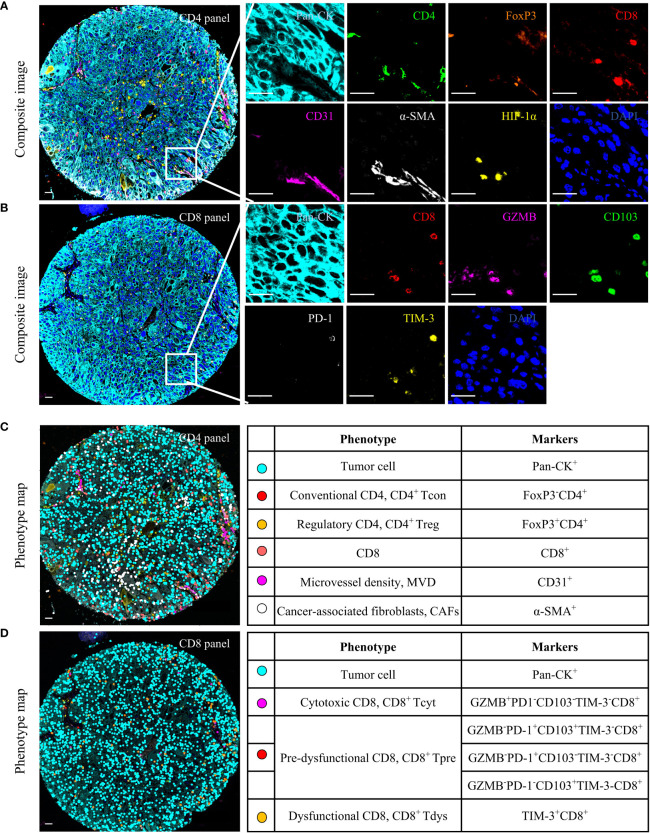
Multiplex immunofluorescence (mIF) analysis of human locally advanced NSCLC. Examples of mIF images **(A, B)** and summary of each defined cell phenotype **(C, D)** and associated markers from the CD4 and CD8 panel. Scale bar, 25 μm.

To obtain multispectral images, the stained slides were scanned at 200x magnification using Vectra Polaris (Akoya Biosciences). A spectral library was used to separate each multispectral image cube into its individual components (spectral unmixing) (InForm 2.4.8, Akoya Biosciences). The InFrom software then automatically recognizes and removes autofluorescence. MIF composited images were shown in **Figures 1A, B**.

### 2.5 Phenotype density analysis

All spectrally unmixed images were subsequently subjected to a proprietary inForm active learning phenotyping algorithm. This allowed for individual identification of each DAPI-stained cell according to their pattern of fluorophore expression and nuclear/cell morphological features. The data exported from the inForm software were further processed using R statistical programming language (version 3.6.3). As shown in **Figures 1C, D**, each functional T cell and tumor cell can associate with specific x and y spatial coordinates. Only tumor cores containing tumor cells in at least 5% of the total tissue area free of artifacts were selected for further analysis.

Both panels included 2-(4-amidinophenyl)-1H-indole-6-carboxamidine (DAPI) as a nuclei marker and Pan-CK to identify tumor cells. CD4 panel aimed to identify the functional status of CD4^+^ T cells, stromal components, and HIF-1α. (i) FoxP3^-^ and FoxP3^+^ were defined as CD4^+^ Tcon and CD4^+^ Treg, respectively, (ii) Two stroma components: CD31 for MVD (22), α-SMA for CAFs (34), and (iii) HIF-1α, a hypoxia marker expressed on tumor cells, CD4 or CD8. CD8 panel was designed to identify three functional states of CD8^+^ T cells. (i) Granzyme B (GZMB) is defined as a cytotoxic CD8 (CD8^+^ Tcyt) (15). (ii) CD103 and PD-1 as pre-dysfunctional markers, and cells expressing one or both of these markers were defined as CD8^+^ Tpre because each exhibited insufficient sensitivity as a single marker (15, 17). (iii) TIM-3 is a marker of CD8^+^ Tdys (15, 17). The counts of phenotypes were normalized to the total cell counts for the total area to generate the density of phenotypes per 1,000 cells.

### 2.6 Spatial interactional analysis

To evaluate the spatial interaction between T cells and tumor cells, we conducted spatial proteomic analyses using the NND and proximity. NND analysis was conducted by calculating the distance (μm) from each tumor cell to its nearest T cell, and proximity analysis was defined as the number of tumor cells with at least one T cell within a 30 μm radius. We selected a 30 μm radius for this study, as it has been previously suggested that these distances represent physiologically plausible distances for direct cell-cell interactions (24, 31, 33, 35, 36).

### 2.7 Statistical analysis

All statistical calculations were performed using IBM SPSS Statistics (version 23.0), GraphPad Prism (version 9.0.0), and R software (version 3.6.3).

The chi-square test or Fisher’s exact test was used to examine differences in categorical variables. The RFS distributions for the patients were estimated using the Kaplan-Meier method. For survival analysis of continuous data, we grouped the patients into high (> cutoff value) or low (≤ cutoff value) groups using survivalROC packages (version 3.6.3), followed by a log-rank test. Regression analysis of the RFS data was performed using the Cox proportional hazards model. Correlations between two parameters were examined using Spearman’s r values and the Kruskal-Wallis test. Statistical significance was set at *P* < 0.05.

## 3 Results

### 3.1 Patient characteristics

**Table 1** list the baseline clinicopathologic characteristics of 99 patients with locally advanced NSCLC that were enrolled in this study. The median age at diagnosis was 59 (range 53-66) years and 68 (69%) patients were men. Smoking index, tumor location, visceral pleural invasion and histology subtype were balanced. The majority of patients had stage IIB/IIIA disease (96%) and underwent adjuvant chemotherapy (88%). The median follow-up time was 37 months (range 3–65). The median RFS time was 28 months (range 1-65). At the time of follow-up, 52 (53%) patients relapsed, and the baseline of relapsed and non-relapsed patients was balanced.

**Table 1:** Patient characteristic.

Variables	Total 99(100%)	Recurrence 52(53%)		Non-Recurrence 47(47%)	*P*
**Age**					1.000
>65y	27 (27)	14 (52)		13 (48)	
≤65y	72 (73)	38 (53)		34 (47)	
**Gender**					0.227
female	31 (31)	13 (42)		18 (58)	
male	68 (69)	39 (57)		29 (43)	
**KPS**					0.407
≥90	60 (61)	29 (48)		31 (52)	
<90	39 (39)	23 (59)		16 (41)	
**Smoking index**					0.084
<400	51 (52)	22 (43)		29 (57)	
≥400	48 (48)	30 (63)		18 (37)	
**Tumor location**					0.924
central	49 (49)	25 (51)		24 (49)	
peripheral	50 (51)	27 (54)		23 (46)	
**Visceral pleural invasion**					1.000
yes	51 (52)	27 (53)		24 (47)	
no	48 (48)	25 (52)		23 (48)	
**Histology subtype**					0.604
SCC	48 (48)	27 (56)		21 (44)	
ADC	51 (52)	25 (49)		26 (51)	
**Pathologic stage**					0.055
IIB	47 (47)	19 (40)		28 (60)	
IIIA	48 (48)	30 (63)		18 (37)	
IIIB	4 (4)	3 (75)		1 (25)	
**Adjuvant chemotherapy**					0.620
yes	87 (88)	47 (54)		40 (46)	
no	12 (12)	5 (42)		7 (58)	
**Adjuvant radiotherapy**					0.609
yes	22 (22)	10 (45)		12 (55)	
no	77 (78)	42 (55)		35 (45)	

Statistical significance determined by two-sided Chi-square test. KPS, Karnofsky performance status; SCC, Squamous cell carcinoma; ADC, Adenocarcinoma.

### 3.2 Characteristics of the locally advanced NSCLC immune microenvironment

**Figure 2:**
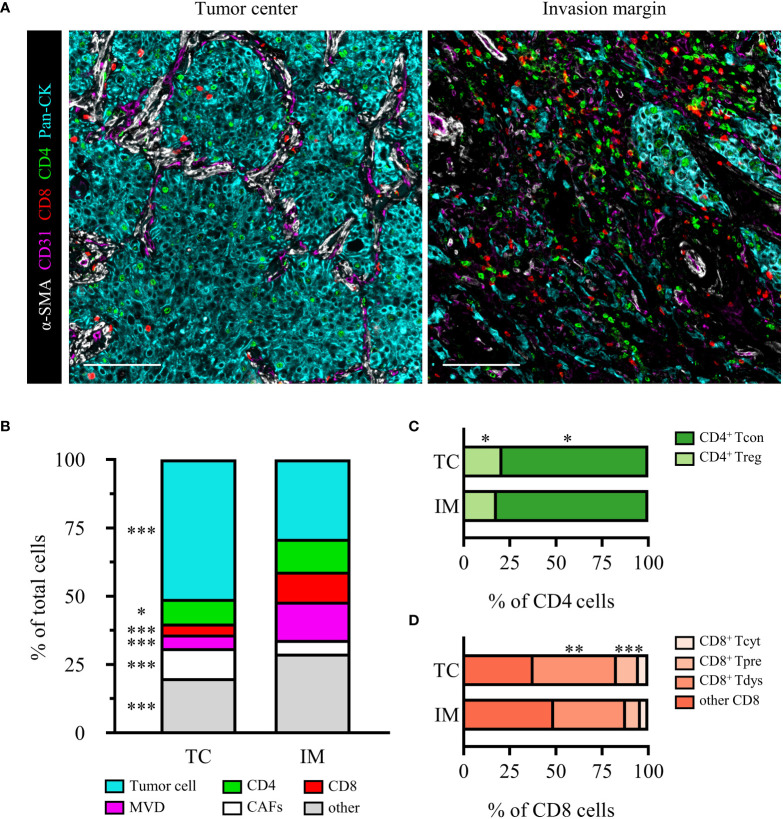
The heterogeneity of TC and IM microenvironment in locally advanced NSCLC. **(A)** Representative composite images of TC and IM from an SCC patient. Scale bar, 100 μm. **(B)** Relative distribution of cell phenotypes in TC and IM. Relative distribution analysis of CD4 **(C)** or CD8 **(D)** subpopulations. Scale bar, 100 μm. Data is presented as the median. *P* values were computed by the Kruskal-Wallis test. *P < 0.05, **P < 0.01, ***P < 0.001.

The heterogeneity of the immune microenvironment between the TC and IM regions was shown in **Figure 2A**. We observed that TC presented with significantly more tumor cells and CAFs than IM, but lower densities of CD4^+^ T cells, CD8^+^ T cells, MVD, and others (**Figure 2B**). CD4^+^ T cells were mainly CD4^+^ Tcon, and compared to TC, the relative proportion of CD4^+^ Tcon in CD4 increased in IM, while CD4^+^ Treg decreased (**Figure 2C**). CD8^+^ T cells were dominated by CD8^+^ Tpre and CD8^+^ Tdys, and the relative proportions of CD8^+^ Tpre and CD8^+^ Tdys within the total CD8^+^ T cells were greater in the TC than in the IM (**Figure 2D**).

### 3.3 The increased density of IM-CD8^+^ Tdys was an independent risk factor for recurrence and was positively correlated with hypoxia

**Figure 3:**
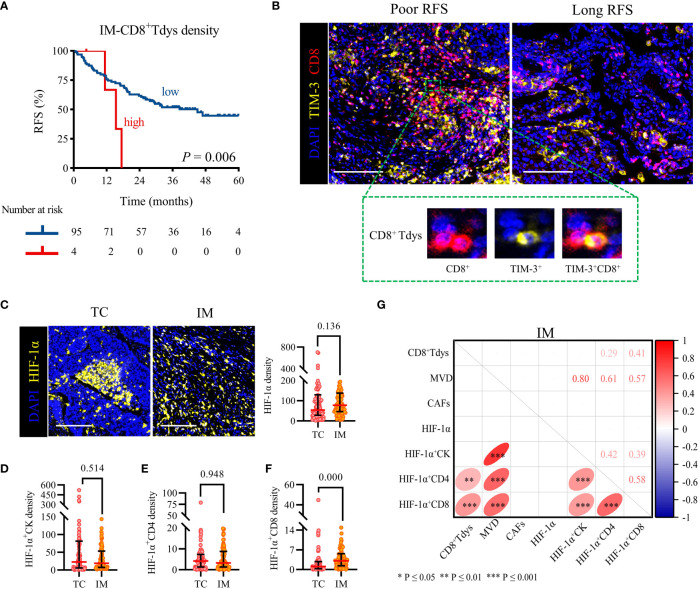
High IM-CD8^+^ Tdys density was associated with the recurrence of the locally advanced NSCLC, and HIF-1α^+^CD8 may contribute to the accumulation of CD8^+^ Tdys. **(A)** Kaplan–Meier curves illustrate the associations between the expression levels of IM-CD8^+^ Tdys (high vs low) and the RFS of locally advanced NSCLC. *P*-values reflect comparisons of two groups by univariate analysis, using the log-rank test. **(B)** Representative image for poor RFS (left) and long RFS (right). Scale bar, 100 μm. **(C–F)** The heterogeneity of HIF-1α **(C)**, HIF-1α^+^CK **(D)**, HIF-1α^+^CD4 **(E)**, and HIF-1α^+^CD8 **(F)** density between TC and IM regions. Scale bar, 100 μm. Significance was determined using the Mann-Whitney test, all data are presented as the median and interquartile ranges. **(G)** Spearman correlation between CD8^+^ Tdys and HIF-1α, MVD, CAFs density in the IM region.

We sought to determine whether the counts of functional T cell subsets were independently correlated with RFS of locally advanced NSCLC. Survival curve analysis showed that CD4^+^ and CD8^+^ T cells infiltration in TC and IM was not linked with RFS (**Supplementary Figures S2A–D**). For their subsets, we observed that only high levels of TC-CD4^+^ Tcon, IM-CD4^+^ Treg, and IM-CD8^+^ Tdys infiltration were associated with shorter RFS (**Supplementary Figures S2E, J** and **Figure 3A**). The Cox proportional hazard regression model was used to evaluate the associations between clinicopathological factors (age, gender, histology subtype, pathologic stage), cell densities (TC-CD4^+^ Tcon, IM-CD4^+^ Treg, IM-CD8^+^ Tdys), and clinical outcome. Only high infiltration of IM-CD8^+^ Tdys was an independent risk factor associated with relapse (*P* = 0.025, HR = 3.536, 95%CI = 1.170-10.690; median RFS, 15.4 vs 45.0 months, *P* = 0.006) (**Table 2** and **Figure 3B**).

**Table 2:** Univariable and Multivariate Cox regression analysis for RFS.

	Univariable		Multivariate	
	*P*	HR (95%CI)	P	HR (95%CI)
Model 1				
Age (≤65 vs. >65)	0.962	1.010(0.550,1.870)	0.709	0.884(0.464,1.685)
Gender (male vs. female)	0.119	1.650(0.879,3.090)	0.341	1.478(0.662,3.299)
Histology subtype (SCC vs. ADC)	0.394	1.270(0.735,2.190)	0.944	0.976(0.493,1.933)
Pathologic stage (IIIA vs. IIB)	0.064	1.720(0.969,3.060)	0.134	1.562(0.871,2.799)
Pathologic stage (IIIB vs. IIB)	0.145	2.480(0.731,8.430)	0.167	2.423(0.691,8.496)
high TC-CD4^+^Tcon density	0.048	3.268(1.011,10.560)	0.053	3.455(0.986,12.107)
high IM-CD4^+^Treg density	0.035	1.916(1.048,3.497)	0.097	1.690(0.910,3.139)
**high IM-CD8^+^Tdys density**	**0.012**	**3.876(1.355,11.050)**	**0.025**	**3.536(1.170,10.690)**
Model 2				
Age (≤65 vs. >65)	0.962	1.010(0.550,1.870)	0.891	1.045(0.558,1.956)
Gender (male vs. female)	0.119	1.650(0.879,3.090)	0.373	1.407(0.663,2.986)
Histology subtype (SCC vs. ADC)	0.394	1.270(0.735,2.190)	0.950	1.021(0.530,1.969)
Pathologic stage (IIIA vs. IIB)	0.064	1.720(0.969,3.060)	0.154	1.532(0.852,2.754)
Pathologic stage (IIIB vs. IIB)	0.145	2.480(0.731,8.430)	0.154	2.513(0.708,8.912)
high TC-CD4^+^Tcon proximity	0.044	1.783 (1.017 ,3.125)	0.416	1.284(0.703,2.347)
**high IM-CD4^+^Treg proximity**	**0.007**	**3.021 (1.344 ,6.757)**	**0.012**	**2.884(1.258,6.615)**

Boldface type indicates statistical significance on multivariate analysis. HR=hazard ratio, CI=confidence interval.

Next, we investigated the correlation between CD8^+^ Tdys and MVD, CAFs, and HIF-1α in the IM region. HIF-1α is not only expressed in tumor cells but also in some immune cells and may affect the infiltration and function of immune cells (37). As shown in **Figures 3C–E**, there was no statistically significant difference in HIF-1α, HIF-1α^+^CK and HIF-1α^+^CD4 density between TC and IM (median, TC vs IM; HIF-1α, 54 vs 78, *P* = 0.136; HIF-1α^+^CK, 23 vs 19, *P* = 0.514; HIF-1α^+^CD4, 4 vs 1, *P* = 0.948), but the density of HIF-1α^+^CD8 in TC was significantly lower than that in IM (1.1 vs 3.1, *P* = 0.000) (**Figure 3F**). Interestingly, the IM-HIF-1α^+^CD8 density was positively correlated with the density of IM-CD8^+^ Tdys (r = 0.41, *P* = 0.000) (**Figure 3G**). Our results suggested that hypoxia may influence the recurrence of locally advanced NSCLC by recruiting CD8^+^ Tdys or promoting CD8^+^ T cells exhaustion.

### 3.4 The nearest neighbor distance of T cells to tumor cells

**Figure 4:**
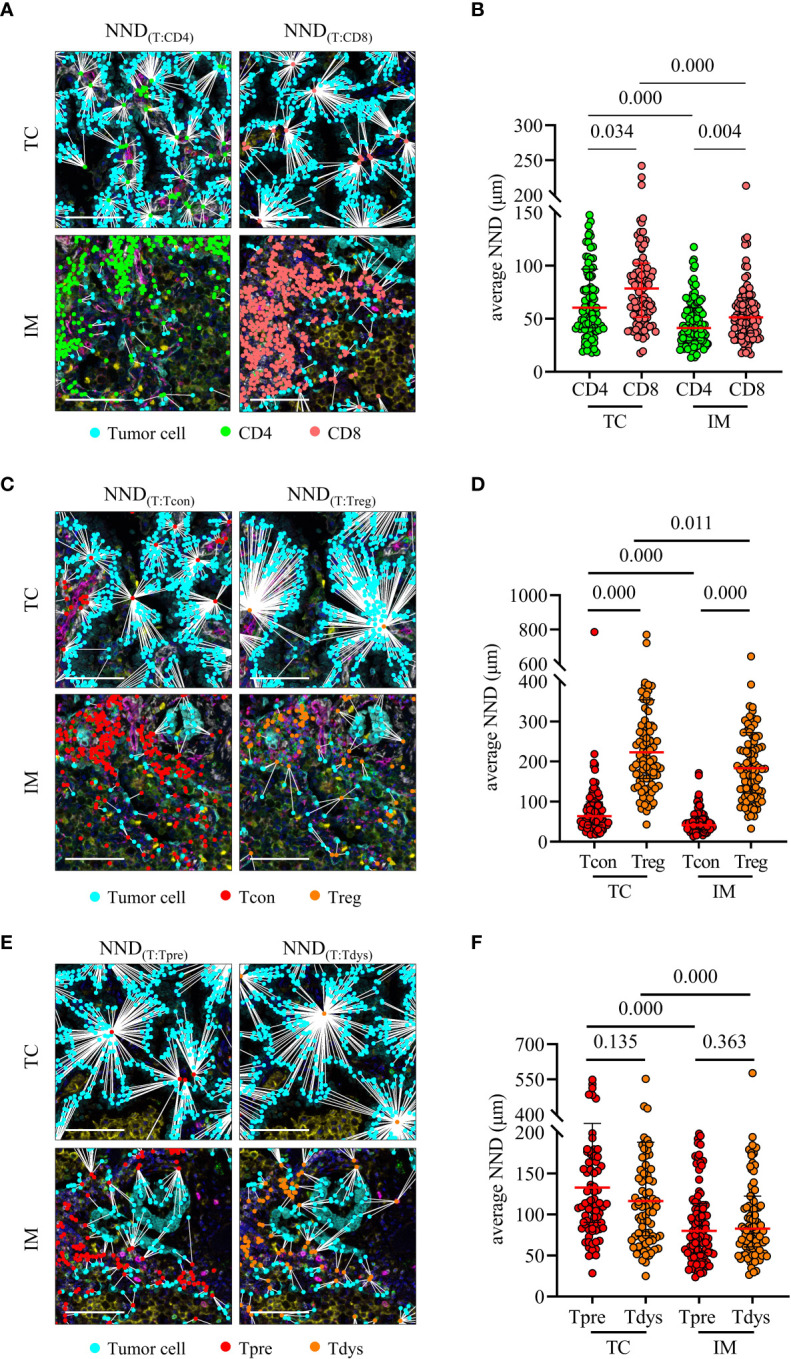
Nearest neighbor distance (NND) analysis of T cells to tumor cells. **(A, B)** NND was calculated from each tumor cell to their nearest CD4 or CD8 (left) and individual value plot of the average NND (right). **(C, D)** NND was calculated from each tumor cell to their nearest CD4^+^ Tcon or CD4^+^ Treg (left) and individual value plot of the average NND (right). **(E, F)** NND was calculated from each tumor cell to their nearest CD8^+^ Tpre or CD8^+^ Tdys (left) and individual value plot of the average NND (right). Scale bar, 50 μm. *P* values were calculated with the Mann-Whitney test, and all data are presented as the median and interquartile ranges.

We analyzed the average nearest neighbor distance between T cells and tumor cells. As shown in **Figures 4A, B**, CD8^+^ T cells were closer to tumor cells than CD4^+^ T cells, both in the TC and IM, and CD4^+^ T cells and CD8^+^ T cells in TC were farther from tumor cells than in the IM. In addition, CD4^+^ Tcon were closer to tumor cells than CD4^+^ Treg, both in the TC and IM; CD4^+^ Tcon and CD4^+^ Treg in TC were farther from tumor cells than in IM (**Figures 4C, D**). Notably, there was no difference in the distance of CD8^+^ Tpre and CD8^+^ Tdys cells from tumor cells, either in TC or IM, CD8^+^ Tpre and CD8^+^ Tdys in TC were farther from tumor cells than in IM (**Figures 4E, F**). These findings led us to hypothesize that the spatial interaction of T cells with tumor cells may represent differences in their biological functions with prognostic significance.

### 3.5 Frequent proximity of CD4^+^ Treg to tumor cells promoted recurrence, and CAFs were positively correlated with their proximity

**Figure 5:**
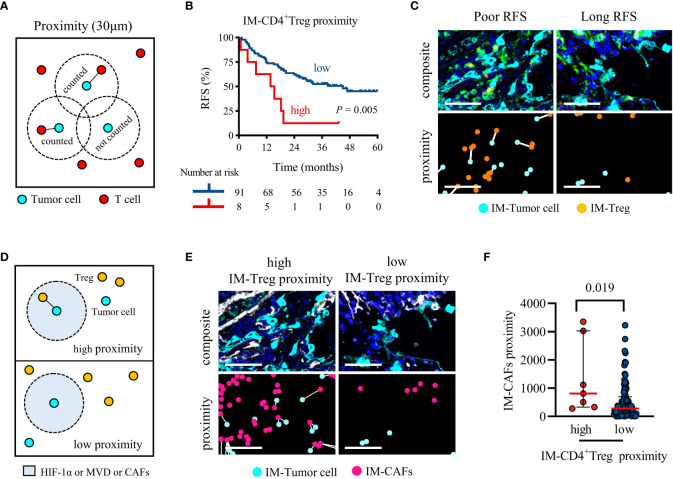
Higher IM-CD4^+^ Treg proximity to tumor cells was associated with significantly shorter RFS, and CAFs recruit CD4^+^ Treg infiltration. **(A)** Schematic diagram of proximity, the black line represents the interaction between tumor cells and T cells. **(B)** Kaplan-Meier survival analyses of the IM-CD4^+^Treg proximity. *P*-values reflect comparisons of two groups by univariate analysis, using the log-rank test. **(C)** Representative image for poor RFS (left) and long RFS (right). Pseudocolor illustrating Pan-CK (cyan), CD4 (green), FoxP3 (orange), and DAPI (blue) staining (composite image). The white line indicates that tumor cells and Treg are within 30 μm of each other (proximity image). Scale bar, 50 μm. **(D)** Schematic representing the parameters analyzed in **(E, F)**. **(E, F)** The proximity of CAFs within 30 μm of cancer cells for each patient separated by cancer cells high or low adjacent CD4^+^ Treg. Pseudocolor illustrating Pan-CK (cyan), α-SMA (white), and DAPI (blue) staining (composite image). The white line indicates that tumor cells and CAFs are within 30 μm of each other (proximity image). Scale bar, 50 μm. *P* values were calculated with the Mann-Whitney test, and all data are presented as the median and interquartile ranges.

Proximity was defined as the number of tumor cells with at least one T cell within a 30 μm radius (**Figure 5A**). High proximity means that frequent interactions are likely to occur. The log-rank test showed that the distance between CD4^+^/CD8^+^ T cells and tumor cells were not related to RFS (**Supplementary Figures S3A–D**). Interestingly, the proximity of TC-CD4^+^ Tcon or IM-CD4^+^ Treg to tumor cells increased and RFS was significantly shortened (**Supplementary Figure S3E** and **Figure 5B**). Multivariate Cox regression analysis showed that only high IM-CD4^+^ Treg proximity was associated with worse RFS (*P* = 0.012, HR = 2.884, 95%CI = 1.258-6.615; median RFS, 14.6 vs 45.0 months, *P* = 0.005) (**Table 2** and **Figure 5C**). In brief, in IM, frequent proximity of CD4^+^ Treg to tumor cells promoted the recurrence of locally advanced NSCLC patients.

As shown in **Figure 5D**, we analyzed the differences in the infiltration of HIF-1α, MVD, and CAFs between high and low IM-CD4^+^ Treg proximity patients. HIF-1α and MVD were not increased in the high IM-CD4^+^ Treg proximity patients (median, high vs low; MVD, 758 vs 574, *P* = 0.071; HIF-1α, 711 vs 346, *P* = 0.071). However, CAFs infiltration was increased (810 vs 277, *P* = 0.019) (**Figures 5E, F**). These results suggested that CAFs may bring CD4^+^ Treg closer to tumor cells in the IM region.

## 4 Discussion

In this study, we comprehensively analyzed the tumor immune microenvironment of TC and IM using TMA and mIF. Our results showed that the distribution of T cells was heterogeneous in the locally advanced TME and the IM region was more immunosuppressed and has a poorer antitumor immune response than the TC region.

T lymphocytes were distributed differently in the TME (7, 25, 26). Therefore, we constructed TC and IM tissue microarrays. To provide satisfactory specimens, two regions of TC and IM of each patient sample were selected to construct the TMA, and the maximum value was used for analysis (28). Our results validated that the density of T cells in IM is higher than that in TC, and we also found that T cells were closer to tumor cells in the IM (7).

Previous studies have shown that the relationship between CD4^+^ and CD8^+^ T cells and RFS was controversial in NSCLC (7, 9, 18, 38). However, when we distinguished the functional status of CD4^+^ and CD8^+^ T cells, only the increased IM-CD8^+^ Tdys density was associated with relapse, further demonstrating the important prognostic value of functional T cell subsets in the IM region. Interestingly, we also found that HIF-1α^+^ CD8 were positively correlated with CD8^+^ Tdys. It has been suggested that hypoxia may promote the exhaustion of CD8 (39). In this study, although we did not find a direct correlation between HIF-1α and CD8^+^ Tdys, our results suggested that HIF-1α^+^ CD8 may promote the exhaustion of CD8. Targeting hypoxia may reduce the presence of CD8^+^ Tdys, thereby sensitizing clinical therapy, the future is worth exploring.

The distance between tumor cells and immune cells might directly reflect the lethality of immune cells toward tumors or, in contrast, the editing of immune cells by tumor cells (35, 40). To the best of our knowledge, no similar studies have been conducted. Our results showed that CD4^+^ and CD8^+^ T cells and their respective subsets were closer to the tumor cells in the IM region. Although the density of IM-CD8^+^ Tdys promoted relapse, the proximity of IM-CD8^+^ Tdys to tumor cells was not associated with recurrence. These results suggested that IM-CD8^+^ Tdys may not interact directly with tumor cells. Notably, we found that the high proximity of IM-CD4^+^ Treg to tumor cells increased the risk of recurrence. A previous study showed that the high proximity of TC-CD4^+^ Treg to tumor cells significantly reduced the OS of lung cancer patients (19). Our study found that it remains important at the invasive margin. Interestingly, CAFs significantly increased in high proximity IM-CD4^+^ Treg patients. A preclinical study on esophageal cancer showed that CAFs may block the infiltration of CD8^+^ T cells and increase the infiltration of CD4^+^ Treg through IL-6, and targeting CAFs can improve the existing tumor immunity and enhance the efficacy of conventional immunotherapy (41). Although no relevant preclinical studies have confirmed the effect of CAFs on the proximity of CD4^+^ Treg to tumor cells in locally advanced NSCLC, our study provided a new direction for targeting CAFs to improve the immunosuppressive microenvironment.

Our study has the following limitations. First, the effective markers of CD8 subsets were not fixed. Future studies in combination with other emerging markers may further reveal the importance of CD8 functional states in NSCLC. Second, our study focused only on CD4^+^ and CD8^+^ T cells, excluding myeloid and other immune cell types. It is necessary to evaluate the importance of other cell types in the NSCLC microenvironment in future studies. In addition, we only found a correlation between HIF-1α, CAFs, and T cells infiltration at the tissue level, which requires further exploration.

In summary, our study highlights the importance of functional status and spatial interaction of T cell subsets, especially in the IM region. Assessment of the density of CD8^+^ Tdys and the proximity of CD4^+^ Treg to tumor cells helped to stratify patients. Targeting HIF-1α and CAFs may provide new treatment strategies for locally advanced NSCLC.

## Data availability statement

The raw data supporting the conclusions of this article will be made available by the authors, without undue reservation.

## Ethics statement

This study was approved by the Ethics Review Committee of Shandong Cancer Hospital and complied with the provisions of the Declaration of Helsinki. This study was a retrospective analysis and informed consent was not required.

## Author contributions

LY, LX and XS contributed to conception and design of the study. LY, WZ, JS, GY, SC and FS organized the database. LY, WZ, JS, GY and SC performed the statistical analysis. LY wrote the first draft of the manuscript. LX and XS wrote sections of the manuscript. All authors contributed to the article and approved the submitted version.
