# Supplementary material for: Functional status and spatial interaction of T cell subsets driven by specific tumor microenvironment correlate with recurrence of non-small cell lung cancer
Source: Front Immunol. 2023 Jan 4;13:1022638. doi: 10.3389/fimmu.2022.1022638 (PMC9846487; doi:10.3389/fimmu.2022.1022638)
Supplement: Supplementary file 1 [file DataSheet_1.docx]

Supplementary Material

# Supplementary Figures


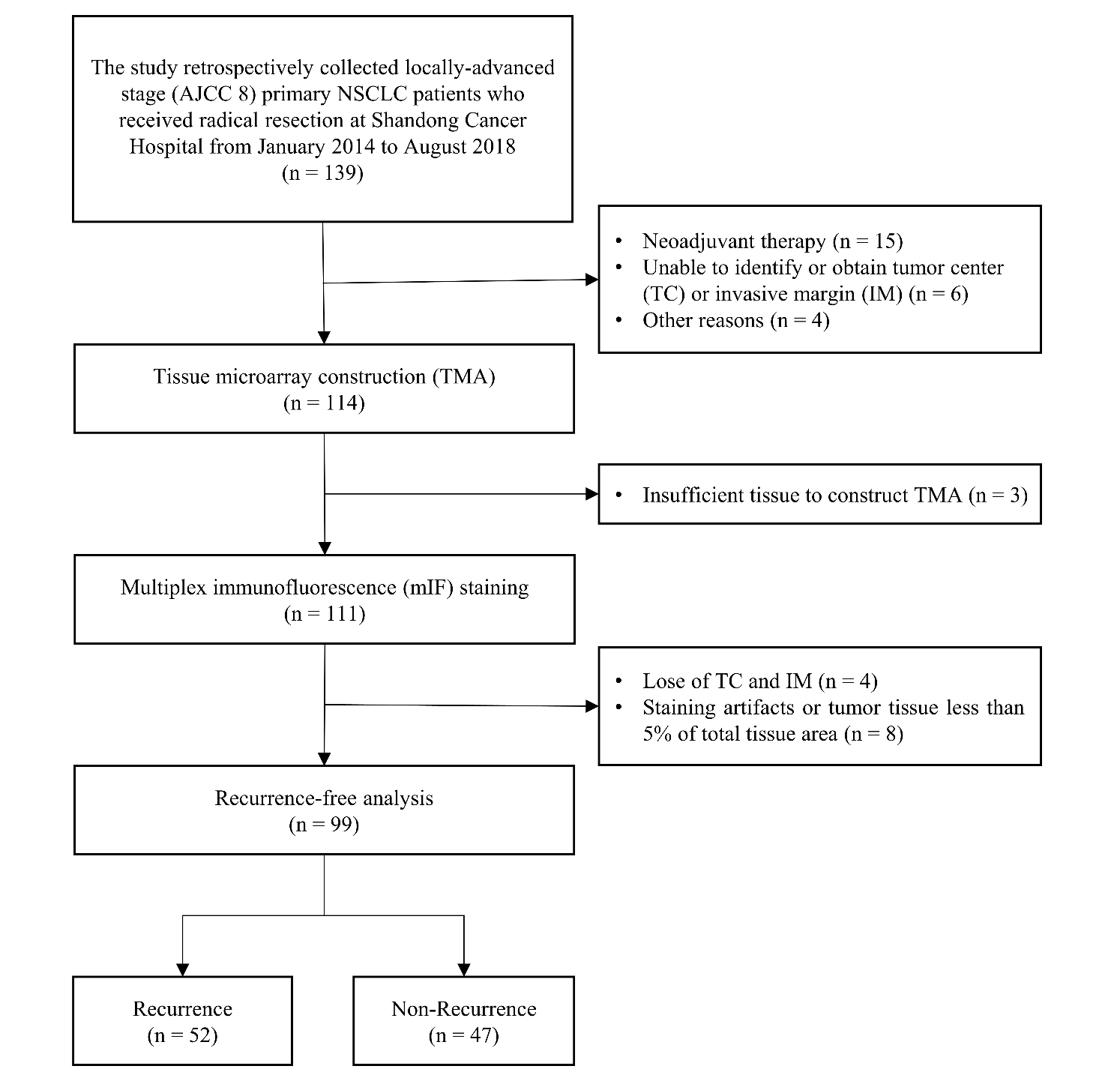


**Supplementary Figure 1.** Study flowchart.

**
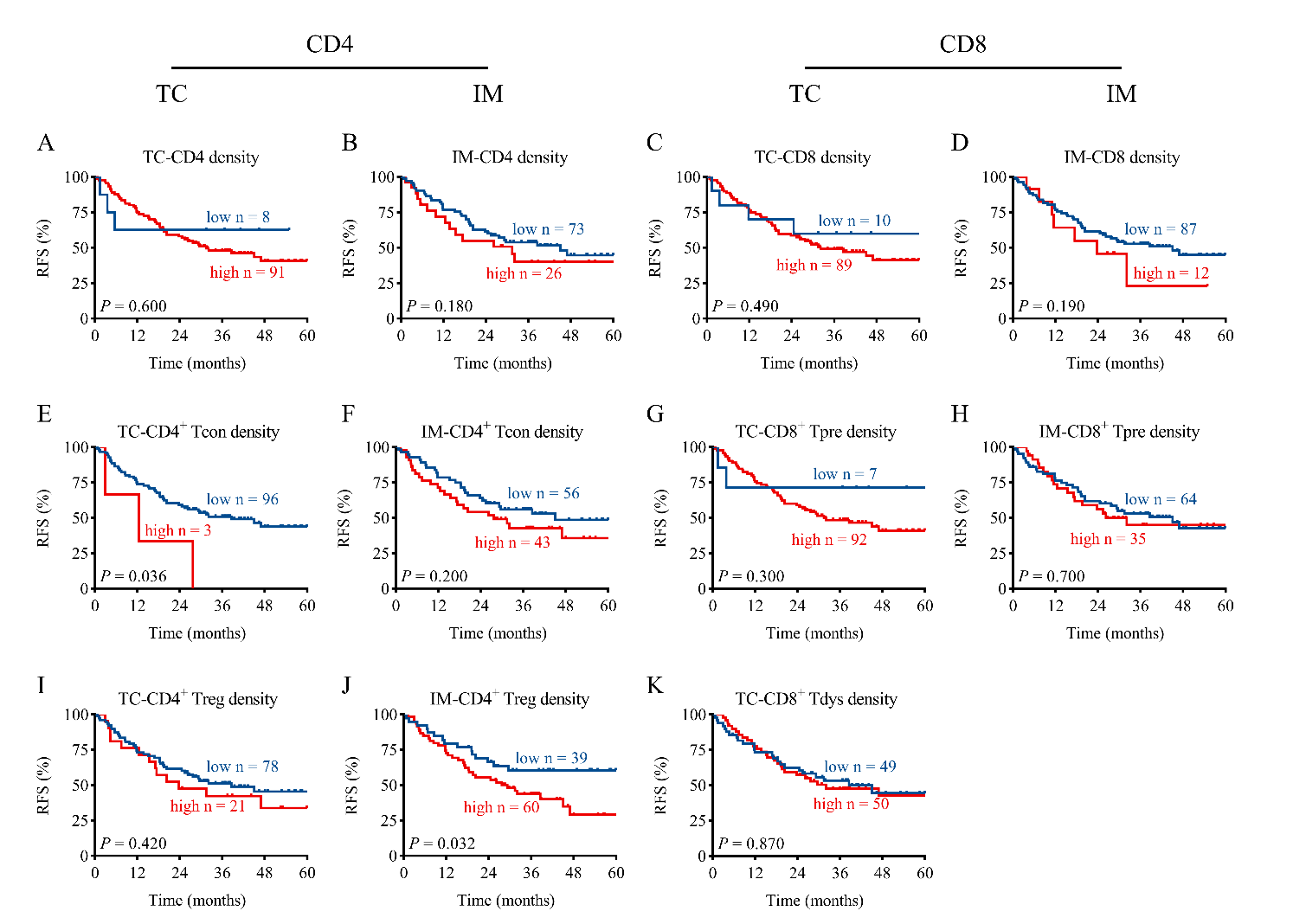
**

**Supplementary Figure 2.** Kaplan–Meier curves illustrate the associations between the expression levels of T cell subsets (high vs low) within the TC and IM areas and the RFS of locally advanced NSCLC. *P*-values reflect comparisons of two groups by univariate analysis, using the log-rank test.

**
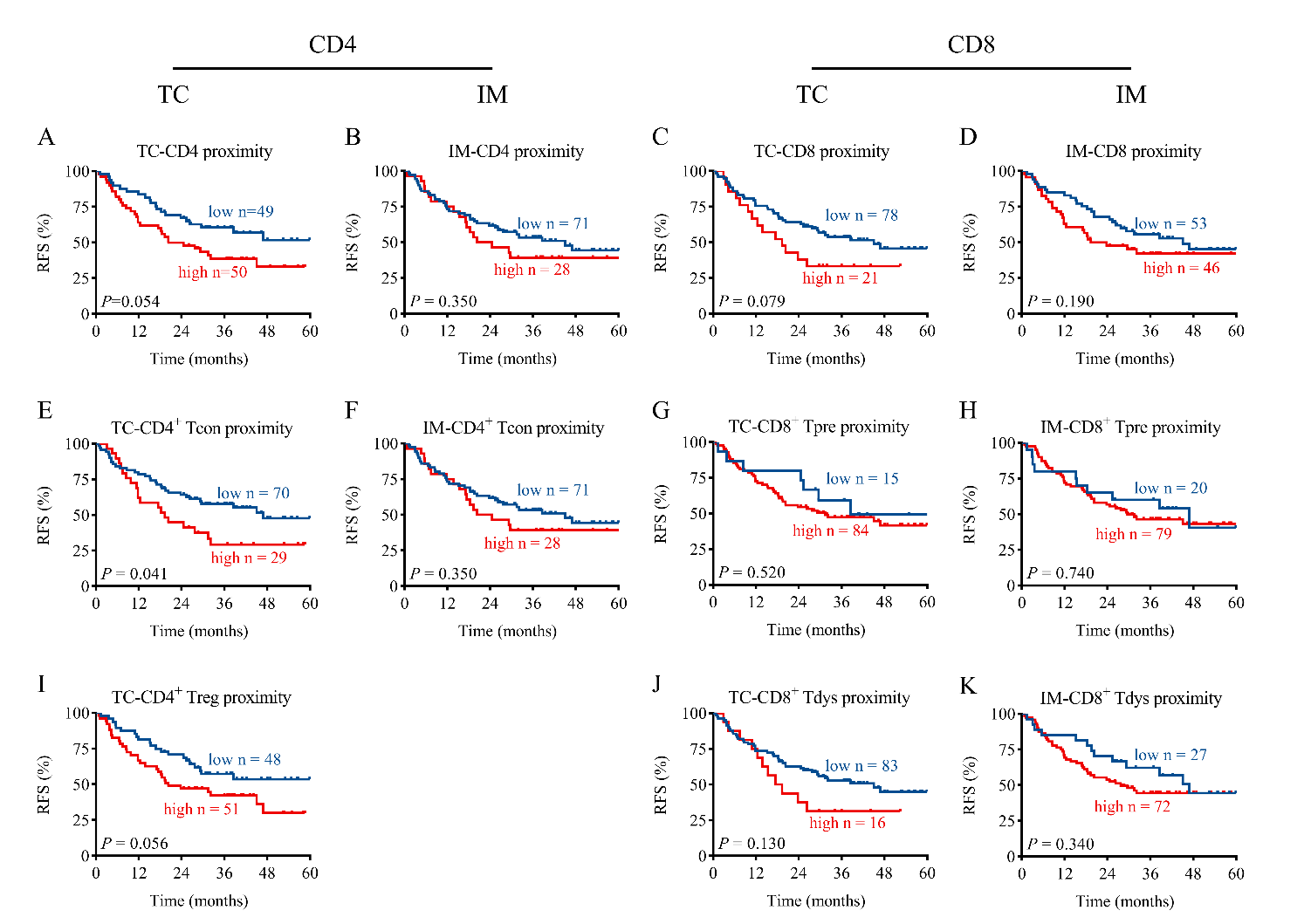
**

**Supplementary Figure 3.** Kaplan–Meier curves illustrate the associations between the proximity of T cells to tumor cells (high vs low) within the TC and IM areas and the RFS of locally advanced NSCLC. *P*-values reflect comparisons of two groups by univariate analysis, using the log-rank test.

# Supplementary Tables

**Supplementary Table S1.** **Antibodies and fluorophores used in the mIF procedures.**

| Panel | Marker | Manufacturer | Dilution | Fluorophore |
| --- | --- | --- | --- | --- |
| CD4 panel | Hif-1α | Abcam, ab51608 | 1:100 | Opal 540 |
|  | CD31 | Abcam, ab76533 | 1:100 | Opal 620 |
|  | Foxp3 | Abcam, ab20034 | 1:100 | Opal 650 |
|  | Pan-CK | Zsbio, ZM-0069 | 1:200 | Opal 480 |
|  | CD8 | Abcam, ab199016 | 1:500 | Opal 690 |
|  | CD4 | Zsbio, ZM-0418 | working fluid | Opal 520 |
|  | α-SMA | Abcam, ab7817 | 1:200 | Opal 570 |
| CD8 panel | CD8 | Abcam, ab199016 | 1:500 | Opal 690 |
|  | GZMB | Abcam, ab255598 | 1:2000 | Opal 540 |
|  | PD-1 | Zsbio, ZM-0069 | 1:200 | Opal 620 |
|  | CD103 | Abcam, ab224202 | 1:200 | Opal 570 |
|  | CK | Zsbio, ZM-0069 | 1:200 | Opal 520 |
|  | TIM-3 | Cell signaling Technology, #45208 | 1:100 | Opal 650 |
